# Olverembatinib (HQP1351)-based therapy in adults with relapsed or refractory Philadelphia chromosome-positive acute lymphoblastic leukemia or chronic myeloid leukemia in blast phase: results from a real-world study

**DOI:** 10.3389/fimmu.2025.1546371

**Published:** 2025-05-14

**Authors:** Ziyu Wen, Zhi Liu, Xu Ye, Zhiping Fan, Ren Lin, Fen Huang, Li Xuan, Xiaofang Li, Hua Jin, Min Dai, Jing Sun, Xuan Zhou, Qiang Wang, Xiaoli Liu, Qifa Liu, Hongsheng Zhou, Na Xu

**Affiliations:** ^1^ Department of Hematology, Nanfang Hospital, Southern Medical University, Guangzhou, China; ^2^ Department of Hematology, Guangdong Second Provincial General Hospital, Guangzhou, China; ^3^ Department of Hematology, Second Affiliated hospital of Guangzhou Medical University, Guangzhou, China; ^4^ Guangdong Provincial Clinical Research Center for Hematologic Diseases, Guangzhou, China

**Keywords:** acute lymphoid leukemia, Philadelphia chromosome positive, Ph^+^, chronic myeloid leukemia, blast phase, olverembatinib, HQP1351

## Abstract

**Background:**

Relapsed or refractory Philadelphia chromosome-positive acute lymphoblastic leukemia (R/R Ph^+^ ALL) and chronic myeloid leukemia in the blast phase (CML-BP) are associated with poor prognoses. Olverembatinib (HQP1351), a novel third-generation tyrosine kinase inhibitor (TKI), has shown promising efficacy and safety in clinical trials against nearly all BCR-ABL1 kinase mutations, including *T315I*.

**Methods:**

Data were collected and analyzed to evaluate the efficacy and safety of olverembatinib-based therapy for advanced Ph^+^ leukemia. The primary outcome was the overall response rate at 28 days. Secondary outcomes included overall survival (OS), event-free survival (EFS), disease-free survival (DFS), the proportion of patients undergoing allo-HSCT, and adverse events.

**Results:**

A total of 59 patients participated in the study, including 40 patients with Ph^+^ ALL and 19 with CML-BP. Among them, 36 (61.0%) were men, and 23 (39.0%) were women. The median age was 39 years (interquartile range [IQR], 30–48), and the median follow-up duration was 7.8 months (IQR, 4.1–11.3). A total of 16 (27.1%) and 11 patients (18.6%) had received treatment with two and ≥ 3 prior TKIs, respectively. Additionally, 19 patients (33.9%) had been treated with ponatinib. In a cohort of 19 CML patients, 12 (63.2%) achieved CR/CRi by day 28. Five (26.3%) achieved a complete cytogenetic response with a median duration of 2.9 months, and two (10.5%) achieved a major molecular response with a median duration of 5.5 months. Among 40 evaluated ALL patients, 37 (92.5%) achieved CR/CRi by day 28, 30 (75.0%) attained MRD negativity, and 22 (55.0%) achieved CMR. The probabilities of DFS, EFS, and OS at 12 months were 80.3% (95% confidence interval [CI]: 61.0%–90.7%), 80.2% (95% CI: 61.0%–90.7%), and 93.3% (95% CI: 75.8%–98.3%) for patients with R/R Ph^+^ ALL, compared to 52.0% (95% CI: 17.7%–78.0%), 23.0% (95%CI, 4.2%-50.6%), and 75.6% (95% CI: 37.7%–92.3%) for those with CML-BP. The prevalent treatment-related nonhematologic adverse events, primarily classified as grade 1/2, included skin hyperpigmentation, proteinuria, increased liver enzyme levels, and hypertriglyceridemia.

**Conclusions:**

Olverembatinib-based therapy demonstrated significant efficacy and manageable toxicity in patients with advanced Ph^+^ leukemia.

## 1 Introduction

Relapsed or refractory Philadelphia chromosome-positive acute lymphoid leukemia (R/R Ph^+^ ALL) and chronic myeloid leukemia (CML) in blast phase (BP) are aggressive leukemias with a poor prognosis (1–4). Tyrosine kinase inhibitors have significantly improved survival and quality of life for patients with Ph^+^ leukemia, particularly third-generation tyrosine kinase inhibitors (TKIs) for those harboring various *BCR::ABL1* mutations (5–8).

Olverembatinib is a novel inhibitor that targets the ATP-binding site of the *BCR::ABL1* kinase. The National Medical Products Administration approved its use in November 2021 for adult chronic or accelerated-phase CML patients harboring the *T315I* mutation. In November 2023, it received approval for a new indication in adult patients with chronic-phase CML who are resistant and/or intolerant to first- and second-generation TKIs (9). Recently, olverembatinib was approved to initiate a global phase III clinical trial and has been included in the latest version of the National Comprehensive Cancer Network guidelines for the treatment of CML (10). The complete remission rate exceeds 90% when frontline treatment for newly diagnosed Ph+ ALL incorporates TKIs in combination with chemotherapy or immunotherapy. However, relapse remains a significant clinical challenge, frequently associated with resistance mutations in the ABL1 kinase domain, particularly the T315I mutation (11–13).

The efficacy and toxicity of an olverembatinib-based regimen in Ph^+^ advanced leukemia remain unclear. Currently, no unified guidelines exist for its treatment strategy. Therefore, we conducted this study to evaluate the activity and safety of olverembatinib in Ph^+^ advanced leukemia.

## 2 Methods

### 2.1 Study design and patients

This retrospective multicenter study was conducted at three hospitals in China—Nanfang Hospital, Guangdong Second Provincial General Hospital, and The Second Affiliated Hospital of Guangzhou Medical University—from December 2021 to October 2023. Data were retrospectively collected and analyzed data from patients aged 14 to 70 years with an Eastern Cooperative Oncology Group performance status of 0 to 2, diagnosed with R/R Ph^+^ ALL or advanced CML according to World Health Organization 2022 classification (14) and the 2020 European LeukemiaNet criteria (15). Exclusion criteria included impaired cardiac function, severe cardiovascular disease, and lactating or pregnant women.

### 2.2 Treatment

Drug doses were administered per prescribed instructions and institutional protocols. Olverembatinib was given at 40 mg orally with meals every 2 days within a 28-day dosing cycle. Treatment continued until disease progression, intolerable toxicity, or other circumstances required cessation of treatment.

Patients diagnosed with myeloid blast phase (MBP) could receive olverembatinib either as monotherapy or in combination with systemic chemotherapy or blinatumomab. Chemotherapy regimens included the HA regimen (homoharringtonine and cytarabine) and hypomethylating agents (HMA) such as azacitidine or decitabine. Patients with lymphoid blast phase (LBP) were treated with either monotherapy or a combination therapy involving the vincristine, daunorubicin, and prednisone (VDP) regimen or blinatumomab. Patients with Ph^+^ ALL could receive olverembatinib in combination with blinatumomab, systemic chemotherapy, or radiotherapy (for extramedullary leukemia only). Chemotherapy regimens included the VDP regimen and the hyper-fractionated cyclophosphamide, vincristine, doxorubicin, and dexamethasone (hyper-CVAD) regimen. Allogeneic hematopoietic stem cell transplantation (allo-HSCT) was considered a potential option after achieving a response if a patient was eligible and a donor was available. Routine intrathecal injection (dexamethasone, 10 mg; cytarabine, 50 mg; and methotrexate, 10 mg) was administered to prevent or treat central nervous system leukemia (16, 17). Eligible patients underwent comprehensive baseline assessments, including a detailed medical history review, physical examination, imaging tests, and laboratory analyses, which covered peripheral blood assessments, bone marrow evaluations, and *BCR::ABL1* transcript analysis.

### 2.3 Assessments

Several outcomes were evaluated, including response rates, overall survival (OS), event-free survival (EFS), disease-free survival (DFS), the proportion of patients undergoing allo-HSCT, and the toxicity of olverembatinib. Complete response (CR) and complete response with incomplete hematological recovery (CRi) were defined as bone marrow blasts < 5% and no extramedullary disease, with or without neutrophil counts < 1.0 × 10^9^/L and platelet counts < 100 × 10^9^/L. OS was defined as the time from the initiation of olverembatinib to death from any cause. EFS was defined as the time from the initiation of olverembatinib to the earliest occurrence of any of the following events: failure at day 28, relapse, treatment discontinuation, or death. DFS was defined as the time from CR/CRi to relapse or death. *BCR::ABL1* transcripts in bone marrow aspirates (when available) or peripheral blood were detected using reverse transcription-quantitative polymerase chain reaction (RT-qPCR), with results expressed on the International Scale (IS). Major molecular response (MMR) and complete molecular response (CMR) were defined as a *BCR::ABL1* transcript level of ≤ 0.1% and undetectable or below the 10^−5^ level by RT-qPCR, respectively. A BCR::ABL1 transcript level of ≤ 1% was considered equivalent to complete cytogenetic remission (CCyR). Measurable residual disease (MRD) negativity was commonly defined using a cutoff of 0.01% by eight-color flow cytometry (18, 19). Additionally, Sanger sequencing was performed in CML patients to assess *BCR::ABL1* mutational status. Adverse events were documented and graded according to the National Cancer Institute Common Terminology Criteria for Adverse Events version 5.0.

### 2.4 Statistical analysis

Descriptive analysis will be used for baseline data and adverse events, reporting numbers and frequencies for qualitative data and medians with interquartile ranges (IQRs) for quantitative data. Kaplan–Meier survival curves were generated for OS, EFS, and DFS. All statistical analyses in this study were conducted using SPSS version 25.0 and GraphPad Prism.

## 3 Patient characteristics

Between 8 December 2021 and 22 October 2023, a total of 59 patients who received olverembatinib-based therapy were included in the study. Among them, 19 patients were diagnosed with CML-BP, including 11 with MBP, seven with LBP, and one with mixed blast phase (MAL), exhibiting both myeloid and lymphoid features. Most patients had previously received TKI treatments, including imatinib, dasatinib, nilotinib, flumatinib, and ponatinib. Patient characteristics are summarized in **Table 1**.

**Table 1:** Patient characteristics.

	Total	Ph^+^ ALL	CML-BP
Patient number	59	40	19
Age (year; median (IQR))	39 (30–48)	38 (28–48)	45 (33–48)
Male (*n* (%))	36 (61.0)	22 (55.0)	14 (73.7)
Female (*n* (%))	23 (39.0)	18 (45.0)	5 (26.3)
ECOG performance status (*n* (%))			
≤ 1	56 (94.9)	38 (95.0)	18 (94.7)
=2	3 (5.1)	2 (5.0)	1 (5.3)
Time from diagnosis to olverembatinib treatment (month; median (IQR))	6.3 (2.5–20.1)	8.2 (2.6-10.8)	20.1 (10.2–87.5)
*BCR::ABL1* transcript (*n* (%))			
p210	32 (54.2)	13 (32.5)	19 (100)
p190	27 (45.8)	27 (67.5)	0 (0)
Number of lines of prior TKI therapy (*n* (%))			
0	10 (16.9)	10 (25.0)*	0 (0)
1	22 (37.3)	20 (50.0)	2 (10.5)
2	16 (27.1)	8 (20.0)	8 (42.1)
3	7 (11.9)	1 (2.5)	6 (31.6)
4	4 (6.8)	1 (2.5)	3 (15.8)
Type of prior TKI therapy (n (%))			
Imatinib	12 (20.3)	3 (7.5)	9 (47.4)
Nilotinib	10 (16.9)	1 (2.5)	9 (47.4)
Dasatinib	41 (69.5)	26 (65.0)	15 (78.9)
Flumatinib	9 (15.3)	3 (7.5)	6 (31.6)
Ponatinib	19 (33.9)	10 (25.0)	9 (47.4)
BCR::ABL1 mutation status (n (%))			
Non-T315I BCR::ABL1 mutation	8 (13.6)	2 (5.0)	6 (31.6)
T315I mutation	9 (15.3)	1 (2.5)	8 (42.1)
Receive HSCT before olverembatinib *n* (%)	12 (20.3)	11 (27.5)	1 (5.3)
With CNSL (n (%))	12 (20.3)	9 (22.5)	3 (15.8)

IQR, interquartile range; ECOG, Eastern Cooperative Oncology Group; TKI, tyrosine kinase inhibitors; HSCT, hematopoietic stem cell transplant; CNSL, central nervous system leukemia.*Ten patients without TKI maintenance therapy post-HSCT experienced disease relapse (Pre-HSCT: 3 imatinib, 7 dasatinib).

### 3.1 CML patients

Among the 19 patients, 14 (73.7%) were men and five (26.3%) were women. The median age was 45 years (IQR: 33–48); the median time from diagnosis to the initiation of olverembatinib was 20.1 months (IQR: 10.2–87.5); and the median follow-up duration was 5.6 months (IQR: 4.1–11.9). All patients exhibited the p210 type of *BCR::ABL1* transcripts. A total of 47.4% (9/19) had received ≥ 3 prior TKIs, while 42.1% (8/19) had received two prior TKIs. Additionally, 47.4% (9/19) had been pretreated with ponatinib, among whom 66.7% developed drug resistance and 33.3% discontinued due to other reasons. Genetic testing revealed that 73.7% (14/19) of patients had mutations. The most prevalent mutations within the *ABL1* kinase domain were *T315I* (42.1%) and *E255K* (15.8%), along with other mutations such as *E255V*, *K357E*, *E462G*, *P1108P*, *D198G*, *M293*, and *H396*. The most common *non-ABL1* kinase domain mutations were *RUNX1* (26.3%) and *ASXL1* (26.3%). Additionally, three (15.8%) patients presented with central nervous system leukemia (CNSL), one (5.3%) had extramedullary lymph node leukemia, and one (5.3%) had undergone allo-HSCT before the initiation of the study.

### 3.2 ALL patients

Among the 40 patients, 22 (55.0%) were men and 18 (45.0%) were women. The median age was 38 years (IQR: 28–48); the median time from diagnosis to initiation of olverembatinib was 8.2 months (IQR: 2.6–10.8); and the median follow-up was 8.3 months (IQR: 5.5–12.5). Patients had either the p210 (13/40; 32.5%) or p190 (27/40; 67.5%) form of *BCR::ABL1* transcripts. A total of eight patients (20.0%) had been treated with two prior TKIs, while two (5.0%) had received ≥ 3. Additionally, 10 patients (25.0%) had been treated with ponatinib, of whom 50.0% developed resistance, 20.0% experienced intolerance, and 30.0% discontinued for other reasons. Three patients had mutations in the *ABL1* kinase domain, including *T315I* (2.5%), *E255K* (2.5%) and *F317L* (2.5%). A total of nine patients (22.5%) had CNSL. Among the 40 patients with Ph^+^ ALL, 26 (65.0%) had primary refractory ALL, while 14 (35.0%) had relapsed refractory ALL, of whom 11 (78.6%) had previously undergone hematopoietic stem cell transplantation.

## 4 Outcomes

**Tables 2**–**4** summarize patient disposition, treatment regimens, and treatment outcomes.

**Table 2:** Patient disposition.

	Total	Ph^+^ ALL	CML-BP
Patient number	59	40	19
Treatment duration month; median (IQR)	7.8 (4.1–11.3)	8.3 (5.5–12.5)	5.6 (4.1–11.9)
Ongoing *n* (%)	37 (62.7)	29 (72.5)	8 (42.1)
Discontinuation *n* (%)	22 (37.3)	11 (27.5)	11 (57.9)
Adverse events	0	0	0
Treatment failure	6 (10.2)	4 (10.0)	2 (10.5)
Death	5 (8.5)	2 (5.0)	3 (15.8)
Other reasons unrelated to efficacy	11 (18.6)	5 (12.5)	6 (31.6)
CR/CRi at day 28 *n* (%)	49 (83.1)	37 (92.5)	12 (63.2)
CR/CRi of pre-ponatinib *n* (%)	14 (73.7)	9 (90.0)	5 (55.6)
CR/CRi, *T315I*-mutated population *n* (%)	5 (55.6)	1 (100.0)	4 (50.0)
MRD negative by FCM	–	30 (75.0)	–
Receive HSCT after CR/CRi *n* (%)	18 (30.5)	13 (32.5)	5 (26.3)
Receive CAR-T after CR/CRi *n* (%)	3 (5.1)	3 (7.5)	0
Receive DLI after CR/CRi *n* (%)	3 (5.1)	3 (7.5)	0

IQR, interquartile range; CR, complete response; CRi, complete response with incomplete hematological recovery; CR/CRi of pre-ponatinib, CR/CRi of patients who had prior ponatinib treatment; MRD, measurable residual disease; HSCT, hematopoietic stem cell transplant; CAR-T, chimeric antigen receptor T cell; DLI, donor lymphocyte infusion.

**Table 3:** Response of patients with CML-BP to olverembatinib alone or in combination with other drugs.

CML-BP	LBP (*n* = 7)	MBP (*n* = 11)	MAL (*n* = 1)	CR/CRi (*n* (%))
HMA	0	2	0	0
HA	0	6	1	5 (71.4)
VDP	4	0	0	3 (75.0)
Blinatumomab	1	0	0	1 (100.0)
Olverembatinib only	2	3	0	3 (60.0)

**Table 4:** Response of patients with Ph^+^ ALL to olverembatinib alone or in combination with other drugs.

Ph^+^ ALL	Primary refractory ALL (*n* = 26)	Relapse with CNSL (*n* = 9)	Relapse without CNSL (*n* = 5)	CR/CRi (*n* (%))
VDP ± venclexta	15	5	2	21 (95.5)
Hyper-CVAD	6	2	1	8 (88.9)
Blinatumomab	5	1	2	7 (87.5)
Radiotherapy	0	1	0	1 (100.0)

### 4.1 CML patients

**Figure 1:**
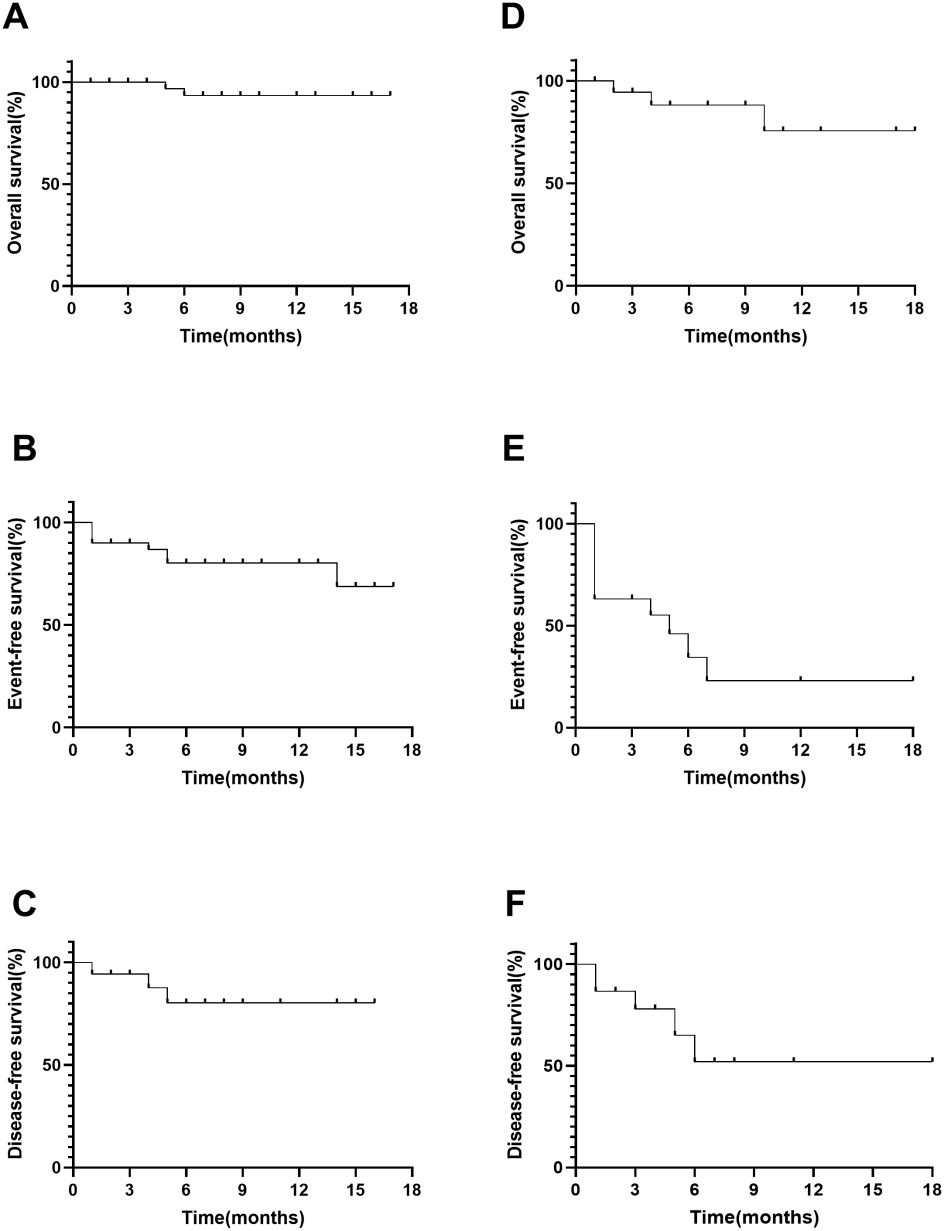
Overall survival, event-free survival, and disease-free survival in ALL **(A–C)** and CML **(D–F)**.

In a cohort of 19 patients with CML-BP, 12 (63.2%) achieved CR/CRi by day 28. Among them, two (10.5%) attained MMR with a median duration of 5.5 months, and five (26.3%) achieved CCyR with a median duration of 2.9 months. Five patients received olverembatinib alone, two were treated in combination with hypomethylating agents, 11 underwent systemic chemotherapy in combination, and one received olverembatinib with blinatumomab. Their CR/CRi rates after 28 days of treatment were 60.0%, 0%, 72.7%, and 100.0%, respectively. All patients who had prior ponatinib treatment received olverembatinib in combination with chemotherapy, achieving a CR/Cri rate of 55.6%. Only one ponatinib-resistant patient attained MMR. A total of five (26.3%) patients underwent allo-HSCT, including one individual with *BCR::ABL*1 transcript levels (IS) > 10%, who experienced relapse within 3 months posttransplantation. The 12-month probabilities of OS, EFS, and DFS in patients with CML-BP were 75.6% (95% confidence interval (CI): 37.7%–92.3%), 23.0% (95%CI, 4.2%-50.6%), and 52.0% (95% CI, 17.7%–78.0%), respectively (**Figure 1**).

### 4.2 ALL patients

In a cohort of 40 patients with Ph^+^ ALL, 37 (92.5%) achieved CR/CRi by day 28, and 30 (75.0%) attained MRD negativity. Additionally, 22 (55.0%), including one patient with the *T315I* mutation, reached CMR. Of these, eight patients received combination therapy with blinatumomab, while 31 underwent combination therapy with chemotherapy, with CR/CRi rates of 87.5% and 93.5%, respectively. Among ponatinib-pretreated patients, seven received combination therapy with chemotherapy, and three received combination therapy with blinatumomab. In this subgroup, 90.0% achieved CR/Cri, and 80.0% attained MMR. After achieving CR/CRi, 13 (32.5%) patients proceeded to undergo allo-HSCT, while three (7.5%) patients with a history of previous transplantation received donor lymphocyte infusion (DLI). Chimeric antigen receptor T-cell (CAR-T) therapy was administered to three patients with BCR::ABL1 transcripts (IS) > 10%; however, one patient experienced relapse 4 months later. The 12-month probabilities of OS, EFS, and DFS in Ph^+^ ALL patients were 93.3% (95% CI: 75.8%–98.3%), 80.2% (95% CI: 61.0%–90.7%), and 80.3% (95% CI: 61.0%–90.7%), respectively (**Figure 1**).

## 5 Safety

Since systemic chemotherapy and immunotherapy can cause myelosuppression, treatment-related hematological adverse events were not documented. With a median follow-up of 7.8 months after olverembatinib initiation, the incidence of adverse events was comparable between the ALL and CML cohorts (**Table 5**). The most commonly reported nonhematologic adverse events were mild (grade 1/2) and resolved either with dosage reduction, supportive therapy, or spontaneously without intervention. Nonhematologic adverse events included skin pigmentation (*n* = 38), proteinuria (*n* = 32), elevated liver enzyme levels (*n* = 25), hypertriglyceridemia (*n* = 24), hyperuricemia (*n* = 8), hyperbilirubinemia (*n* = 8), rash (*n* = 6), arterial occlusion events (*n* = 2), and elevated creatine kinase (*n* = 1).

**Table 5:** Treatment-related adverse events.

No. (%)	Total (*n* = 59)		ALL (*N* = 40)		CML (*N* = 19)	
Nonhematological AEs	Any grade	Grade 3/4	Any grade	Grade 3/4	Any grade	Grade 3/4
Skin pigmentation	38 (64.4)	0	30 (75.0)	0	8 (42.1)	0
Proteinuria	32 (54.2)	0	24 (60.0)	0	8 (42.1)	0
Elevated liver enzyme	25 (42.4)	4 (6.8)	18 (45.0)	3 (7.5)	7 (36.8)	1 (5.3)
Hypertriglyceridemia	24 (40.7)	2 (3.4)	21 (52.5)	2 (5.0)	3 (15.8)	0
Hyperuricemia	8 (13.6)	1 (1.7)	7 (17.5)	1 (2.5)	1 (5.3)	0
Hyperbilirubinemia	8 (13.6)	1 (1.7)	5 (12.5)	1 (2.5)	3 (15.8)	0
Rash	6 (10.2)	0	5 (12.5)	0	1 (5.3)	0
Arterial occlusion event	2 (3.4)	1 (1.7)	2 (5.0)	1 (2.5)	0	0
Elevated creatine kinase	1 (1.7)	1 (1.7)	0	0	1 (5.3)	1 (5.3)

Treatment-related hematological adverse events were not documented.

In the study, no patients experienced treatment-related pancreatitis, hypertension, arrhythmia, prolonged QT interval, pleural effusion, pericardial effusion, or gastrointestinal adverse events such as diarrhea and constipation. Among all patients, 37 (62.7%) continued treatment, while 22 (37.3%) discontinued due to disease progression, intolerance, loss to follow-up, death, or other reasons.

## 6 Discussion

This study demonstrated that olverembatinib-based therapy exhibited significant efficacy in heavily pretreated patients with advanced Ph^+^ leukemia who had developed resistance to multiple TKIs.

The prognosis for newly diagnosed CML-BP patients or those progressing from CP/AP to BP is notably poor, with a median overall survival of less than 1-year postdiagnosis (20–23). Third-generation TKIs have demonstrated the ability to achieve deeper responses than first- and second-generation TKIs and effectively overcome resistance (9, 24–26). Olverembatinib is the only third-generation TKI available in China. However, research on the efficacy of olverembatinib-based therapy in advanced CML remains limited. Jiang et al. reported that among 38 patients with TKI-resistant CML-AP treated with olverembatinib, 18 achieved MCyR and CCyR at a median time of 3 months (range, 1–9) and 4 months (range, 1–15), respectively. The rates of MMR and MR4.0 were 44.7% and 36.8%, respectively (24). In this study, 12 (63.2%) patients with CML-BP achieved CR/CRi by day 28. Additionally, two (10.5%) reached MMR at a median time of 5.5 months, while five (26.3%) reached CCyR at a median time of 2.9 months. The 12-month probabilities of OS, EFS, and DFS in patients with CML-BP were 75.6% (95% CI: 37.7%–92.3%), 23.0% (95%CI, 4.2%-50.6%) and 52.0% (95% CI: 17.7%–78.0%), respectively. Despite nearly half of the CML patients in our study having prior treatment with ponatinib and 3 (15.8%) presenting with CNSL, our outcomes closely aligned with those reported in the studies by Jiang et al. and the PACE trial (24, 26). These findings suggest that olverembatinib-based treatment is a potent and effective induction therapy for patients with CML-BP.

Olverembatinib more effectively inhibits p-KIT, p-AKT, p-ERK1/2, and p-STAT3 than ponatinib, targeting markers highly expressed in hematological malignancies. It suppresses pre-B ALL cells by inhibiting SRC kinase and the PI3K/AKT pathways, highlighting its potential as a therapeutic agent for pre-B ALL (27–29). A recent study by Jabbour et al. (25) demonstrated the promising efficacy of olverembatinib in patients with heavily pretreated or refractory CML and Ph^+^ ALL outside of China. In the evaluable cohort of ponatinib-failed patients, 53.3% attained CCyR and 37.5% achieved MMR in the CML-CP group, while 28.6% attained CCyR and 22.2% achieved MMR in the ALL group. In our study, among ponatinib-pretreated patients, the rates of MMR were 80.0% in the ALL cohort and 11.1% in the CML-BP cohort. Compared to the study by Jabbour et al., outcomes in the ALL cohort appear more favorable, whereas those in the CML cohort are less satisfactory. One possible explanation for this discrepancy is that most Ph^+^ ALL patients who had failed third-generation TKI therapy in Jabbour’s study were treated exclusively with olverembatinib, whereas the CML patients included were in the chronic phase. Collectively, these findings support the potential of olverembatinib as a highly effective therapeutic backbone agent for patients with Ph^+^ ALL resistant to multiple TKIs.

Xiang et al. successfully detected olverembatinib concentrations in the patient’s cerebrospinal fluid (CSF) (30). Li et al. also confirmed that in pediatric patients with relapsed refractory ALL and CNSL, the addition of olverembatinib can rapidly eliminate leukemia blast cells in CSF (31). In this study, eight of 12 patients with CNSL achieved CSF negativity following olverembatinib-based treatments, further suggesting a potential role for olverembatinib in managing CNSL in patients with Ph^+^ leukemia.

Common AEs in patients receiving olverembatinib therapy are predominantly hematological, dermatological, or associated with proteinuria and abnormal biochemical indicators (9, 24). The most common nonhematological AEs were elevated liver enzymes, proteinuria and skin pigmentation. Jiang et al. reported a 32.0% incidence of cardiovascular events related to olverembatinib during a median follow-up of 3 years, with arterial occlusive and venous thrombotic events occurring in 5.0% of patients—lower than the 31.0% reported for ponatinib over a 5-year median follow-up (24). This study reported that two patients (3.4%) experienced arterial occlusion events, including cerebral infarction and pulmonary embolism. Of note, both patients had prior exposure to ponatinib. Nonetheless, vigilant monitoring remains essential for cardiovascular thrombotic events associated with olverembatinib.

This study has several limitations: the heterogeneity of patient populations and treatment regimens limits the ability to draw general conclusions; its retrospective nature precludes a detailed assessment of treatment-related adverse events; and the small sample size hinders a comprehensive evaluation of the cardiovascular toxicity of olverembatinib. However, a key strength of observational studies is their relevance to real-world patient populations.

## 7 Conclusion

In summary, these findings demonstrate that the olverembatinib-based regimen shows favorable efficacy and safety in patients with CML-BP and Ph^+^ ALL. Prospective randomized controlled clinical trials with larger and more diverse patient populations are needed to confirm these results.

## Data Availability

The original contributions presented in the study are included in the article/supplementary material. Further inquiries can be directed to the corresponding authors.

## References

[1] Balsat M Cacheux V Carre M Tavernier-Tardy E Thomas X. Treatment and outcome of Philadelphia chromosome-positive acute lymphoblastic leukemia in adults after relapse. Expert Rev Anticancer Ther. (2020) 20:879–91. 10.1080/14737140.2020.1832890 33016157

[2] Senapati J Jabbour E Kantarjian H Short NJ. Pathogenesis and management of accelerated and blast phases of chronic myeloid leukemia. Leukemia. (2023) 37:5–17. doi: 10.1038/s41375-022-01736-5 36309558

[3] Copland M. Treatment of blast phase chronic myeloid leukaemia: A rare and challenging entity. Br J haematol. (2022) 199:665–78. 10.1111/bjh.18370 PMC9796596 35866251

[4] Perrone S Massaro F Alimena G Breccia M. How has treatment changed for blast phase chronic myeloid leukemia patients in the tyrosine kinase inhibitor era? A review of efficacy and safety. Expert Opin pharmacother. (2016) 17:1517–26. doi: 10.1080/14656566.2016.1190335 27231757

[5] Cross SA Lyseng-Williamson KA. Imatinib: in relapsed or refractory Philadelphia chromosome-positive acute lymphoblastic leukaemia. Drugs. (2007) 67:2645–54. doi: 10.2165/00003495-200767170-00013 18034597

[6] Jabbour E Short NJ Jain N Huang X Montalban-Bravo G Banerjee P. Ponatinib and blinatumomab for Philadelphia chromosome-positive acute lymphoblastic leukaemia: a US, single-centre, single-arm, phase 2 trial. Lancet Haematol. (2023) 10:e24–34. doi: 10.1016/S2352-3026(22)00319-2 36402146

[7] Couturier MA Thomas X Raffoux E Huguet F Berthon C Simand C. Blinatumomab + ponatinib for relapsed/refractory Philadelphia chromosome-positive acute lymphoblastic leukemia in adults. Leukemia lymphoma. (2021) 62:620–9. doi: 10.1080/10428194.2020.1844198 33153370

[8] Tavitian S Uzunov M Bérard E Bouscary D Thomas X Raffoux E. Ponatinib-based therapy in adults with relapsed or refractory Philadelphia chromosome-positive acute lymphoblastic leukemia: results of the real-life OPAL study. Leukemia lymphoma. (2020) 61:2161–7. doi: 10.1080/10428194.2020.1762876 32508181

[9] Dhillon S. Olverembatinib: first approval. Drugs. (2022) 82:469–75. doi: 10.1007/s40265-022-01680-9 35195876

[10] Shah NP Bhatia R Altman JK Amaya M Begna KH Berman E. Chronic myeloid leukemia, version 2.2024, NCCN clinical practice guidelines in oncology. J Natl Compr Cancer Network: JNCCN. (2024) 22:43–69. doi: 10.6004/jnccn.2024.0007 38394770

[11] Wassmann B Pfeifer H Goekbuget N Beelen DW Beck J Stelljes M. Alternating versus concurrent schedules of imatinib and chemotherapy as front-line therapy for Philadelphia-positive acute lymphoblastic leukemia (Ph+ ALL). Blood. (2006) 108:1469–77. doi: 10.1182/blood-2005-11-4386 16638934

[12] Mizuta S Matsuo K Yagasaki F Yujiri T Hatta Y Kimura Y. Pre-transplant imatinib-based therapy improves the outcome of allogeneic hematopoietic stem cell transplantation for BCR-ABL-positive acute lymphoblastic leukemia. Leukemia. (2011) 25:41–7. doi: 10.1038/leu.2010.228 20944676

[13] Soverini S Bassan R Lion T. Treatment and monitoring of Philadelphia chromosome-positive leukemia patients: recent advances and remaining challenges. J Hematol Oncol. (2019) 12:39. doi: 10.1186/s13045-019-0729-2 31014376 PMC6480772

[14] Khoury JD Solary E Abla O Akkari Y Alaggio R Apperley JF. The 5th edition of the world health organization classification of haematolymphoid tumours: myeloid and histiocytic/dendritic neoplasms. Leukemia. (2022) 36:1703–19. doi: 10.1038/s41375-022-01613-1 PMC9252913 35732831

[15] Hochhaus A Baccarani M Silver RT Schiffer C Apperley JF Cervantes F. European LeukemiaNet 2020 recommendations for treating chronic myeloid leukemia. Leukemia. (2020) 34:966–84. doi: 10.1038/s41375-020-0776-2 PMC7214240 32127639

[16] Jabbour EJ Faderl S Kantarjian HM. Adult acute lymphoblastic leukemia. Mayo Clinic Proc. (2005) 80:1517–27. doi: 10.4065/80.11.1517 16295033

[17] Sancho JM Ribera JM Oriol A Hernandez-Rivas JM Rivas C Bethencourt C. Central nervous system recurrence in adult patients with acute lymphoblastic leukemia: frequency and prognosis in 467 patients without cranial irradiation for prophylaxis. Cancer. (2006) 106:2540–6. doi: 10.1002/cncr.v106:12 16700036

[18] Campana D. Minimal residual disease in acute lymphoblastic leukemia. Hematol Am Soc Hematol Educ Program. (2010) 2010:7–12. doi: 10.1182/asheducation-2010.1.7 21239764

[19] Kruse A Abdel-Azim N Kim HN Ruan Y Phan V Ogana H. Minimal residual disease detection in acute lymphoblastic leukemia. Int J Mol Sci. (2020) 21. doi: 10.3390/ijms21031054 PMC7037356 32033444

[20] Jain P Kantarjian HM Ghorab A Sasaki K Jabbour EJ Nogueras Gonzalez G. Prognostic factors and survival outcomes in patients with chronic myeloid leukemia in blast phase in the tyrosine kinase inhibitor era: Cohort study of 477 patients. Cancer. (2017) 123:4391–402. doi: 10.1002/cncr.v123.22 PMC5673547 28743165

[21] Maiti A Franquiz MJ Ravandi F Cortes JE Jabbour EJ Sasaki K. Venetoclax and BCR-ABL tyrosine kinase inhibitor combinations: outcome in patients with philadelphia chromosome-positive advanced myeloid leukemias. Acta Haematol. (2020) 143:567–73. doi: 10.1159/000506346 PMC7839068 32289808

[22] Deau B Nicolini FE Guilhot J Huguet F Guerci A Legros L. The addition of daunorubicin to imatinib mesylate in combination with cytarabine improves the response rate and the survival of patients with myeloid blast crisis chronic myelogenous leukemia (AFR01 study). Leukemia Res. (2011) 35:777–82. doi: 10.1016/j.leukres.2010.11.004 21145590

[23] Abaza Y Kantarjian H Alwash Y Borthakur G Champlin R Kadia T. Phase I/II study of dasatinib in combination with decitabine in patients with accelerated or blast phase chronic myeloid leukemia. Am J Hematol. (2020) 95:1288–95. doi: 10.1002/ajh.v95.11 PMC12375286 32681739

[24] Jiang Q Li Z Qin Y Li W Xu N Liu B. Olverembatinib (HQP1351), a well-tolerated and effective tyrosine kinase inhibitor for patients with T315I-mutated chronic myeloid leukemia: results of an open-label, multicenter phase 1/2 trial. J Hematol Oncol. (2022) 15:113. doi: 10.1186/s13045-022-01334-z 35982483 PMC9389804

[25] Jabbour E Kantarjian HM Koller PB Jamy O Oehler VG Lomaia E. Update of olverembatinib (HQP1351) overcoming ponatinib and/or asciminib resistance in patients (Pts) with heavily pretreated/refractory chronic myeloid leukemia (CML) and philadelphia chromosome-positive acute lymphoblastic leukemia (Ph + ALL). Blood. (2023) 142(Supplement 1):1798. doi: 10.1182/blood-2023-187744

[26] Cortes JE Kim DW Pinilla-Ibarz J le Coutre P Paquette R Chuah C. A phase 2 trial of ponatinib in Philadelphia chromosome-positive leukemias. New Engl J Med. (2013) 369:1783–96. doi: 10.1056/NEJMoa1306494 PMC3886799 24180494

[27] Wang Y Zhang L Tang X Luo J Tu Z Jiang K. GZD824 as a FLT3, FGFR1 and PDGFRα Inhibitor against leukemia *in vitro* and *in vivo* . Trans Oncol. (2020) 13:100766. doi: 10.1016/j.tranon.2020.100766 PMC7125355 32247263

[28] Ren X Pan X Zhang Z Wang D Lu X Li Y. Identification of GZD824 as an orally bioavailable inhibitor that targets phosphorylated and nonphosphorylated breakpoint cluster region-Abelson (Bcr-Abl) kinase and overcomes clinically acquired mutation-induced resistance against imatinib. J medicinal Chem. (2013) 56:879–94. doi: 10.1021/jm301581y 23301703

[29] Zhang T Zhou H Xu M Qian C Sun A Wu D. Combination venetoclax and olverembatinib (HQP1351) as a successful therapeutic strategy for relapsed/refractory (R/R) mixed-phenotype blast phase of chronic myeloid leukemia. Ann hematol. (2023) 102:973–5. doi: 10.1007/s00277-023-05110-y 36745193

[30] Xiang D Zhao T Wang J Cao Y Yu Q Liu L. Determination of olverembatinib in human plasma and cerebrospinal fluid by an LC-MS/MS method: Validation and clinical application. J Pharm Biomed analysis. (2023) 230:115382. doi: 10.1016/j.jpba.2023.115382 37060798

[31] Li X Zhang J Liu F Liu T Zhang R Chen Y. Olverembatinib treatment in pediatric patients with relapsed philadelphia-chromosome-positive acute lymphoblastic leukemia. Clin lymphoma myeloma leukemia. (2023) 23:660–6. doi: 10.1016/j.clml.2023.04.012 37301632

